# Novel pathogenic mutations in the glucocerebrosidase locus

**DOI:** 10.1016/j.ymgme.2012.05.006

**Published:** 2012-08

**Authors:** Raquel Duran, Alisdair McNeill, Atul Mehta, Derralyn Hughes, Timothy Cox, Patrick Deegan, Anthony H.V. Schapira, John Hardy

**Affiliations:** a Reta Lilla Weston Laboratories and Departments of Molecular Neuroscience, UCL Institute of Neurology, Queen Square, London WC1N 3BG, UK; b Department of Clinical Neurosciences, Institute of Neurology, UCL Royal Free Campus Medical School, Pond Street, Pond Street, London NW3 2PF, UK; c Lysosomal Storage Disorders Unit, Department of Haematology, Royal Free Hospital, London, UK; d Lysosomal Diseases Unit, Addenbrookes Hospital, and Department of Medicine, University of Cambridge, UK

**Keywords:** Parkinson's disease, Genetics, Gaucher's disease, Glucocerebrosidase, GBA1 gene

## Abstract

To determine the frequency of mutations responsible for Gaucher's disease, we systematically sequenced the *GBA1* gene as part of a molecular characterization of 73 adult patients in the United Kingdom. Five hitherto unknown pathogenic variants were identified, one of which is a splice site change; the others are novel missense mutations. Given that *GBA1* gene mutations are an important risk factor for the development of Parkinson's disease, we contend that a complete analysis and molecular characterization of both the known and novel *GBA1* variants will be needed before the biochemical processes underlying this genetic association can be fully understood.

## Highlights

► We report a comprehensive genotypic analysis of *GBA1* in 73 Type I GD patients. ► We identified 5 new mutations in the *GBA1* gene. ► The mutations we report here are clearly loss of function alleles.

## 1 Introduction

The identification of the glucocerebrosidase locus (*GBA1*) as a strong genetic determinant of Parkinson's disease (PD) [1], renders it difficult to counsel patients with Gaucher's disease (GD) and their relatives as to their lifetime risk of this disorder. Furthermore, as more patients with PD are undergoing genetic analysis, *GBA1* mutations of unknown significance are increasingly being found in this population. Impaired activity, function or altered processing of glucocerebrosidase causes GD [2] but the relationship of individual mutant *GBA1* alleles and their effects on the structure and activity of the cognate β-glucocerebrosidase to the development of parkinsonism has yet to be determined. As part of a nationwide study of the pathogenesis of parkinsonian manifestations in adult GD patients, we have undertaken a systematic approach to the molecular characterization of the *GBA1* gene as a contribution to extend the list of GD causing alleles and a previous step towards the better understanding of the functional consequences of the mutant protein which would underlie this association.

## 2 Materials and methods

### 2.1 Patients

Seventy-three consecutive unrelated adult patients with Type I GD were recruited from specialised clinics for patients with lysosomal diseases at the Royal Free Hospital, London, and Addenbrooke's Hospital, Cambridge. Thirty-nine (53%) were male, mean age was 50 (range 18–89 years), 16 were Ashkenazim (22%) and 4 had Eastern European ancestry (5.7%); the remainder were white UK citizens without known Ashkenazim ancestry. Genomic DNA was extracted from lymphocytes or saliva. Exons 1–11 of the *GBA1* gene were sequenced using a published protocol [3]. The sequence reference and exon numbering were those of GenBank accession number NM_000157.3. Written informed consent was taken from each participant; this study was approved by the North West London and Cambridge University Hospitals ethics committees.

## 3 Results

### 3.1 Mutations in GBA1 in Type I (‘non-neuronopathic’) GD patients

One hundred and forty four mutant *GBA1* alleles were identified in the patients, of these 139 had been described. In two patients only a single mutant allele was found. Fifty percent were N370S (n = 74), 12% were L444P (n = 19), 6.0% were RecNcil (n = 8), 2.7% were R463C (n = 4), 2% D409H (n = 3) and 1.3% were 84GG, R120W, M123T, S173X, G202R, W184R or R496H (two for each). The following known disease-associated mutations were found as a single copy in GD patients: IVS2 + 1G > A, 155-156insACAGCT, 203insC, 329-333delCAGAA, L66P, R120Q, P182T, F216Y, H255Q, P266R, A318D, R359X, D380N, D380A, 1263-1317del55, L480P. In two patients a second mutation could not be identified, leaving genotype assignments as: R496H/not found and N370S/not found, in patients with a clear enzymatic deficiency and a clinical diagnosis fully consistent with ‘non-neuronopathic’ GD. When genotype does not correspond with clinical phenotype, DNA rearrangements such as deletions, fusions and/or duplications have to be considered as a consequence of recombination events. Appropriate methods are being optimized in our laboratory to investigate these possibilities.

### 3.2 Five novel GBA1 mutations identified in U.K Type I GD patients

We identified 5 mutations in the *GBA1* gene that were hitherto unknown since they have not been reported in 2500 chromosomes sequenced by 1000 Genomes Project (http://www.1000genomes.org/): G250V (c.866G > T) (found trans to N370S), R262G (c.901C > G) (found trans to RecNil), A341V (c.1139G > A) (found trans to N370S ), IVS9 + 1G > A (c.1388 + 1G > A) (found trans to N370S in two patients) and V447E (c.1457T > A) (found trans to N370S). The clinical features of the subjects harbouring these mutations are summarised in Table 1. Their GBA1 enzymatic activity values were < 15% of control values consistent with a diagnosis of GD. No cognitive impairment or parkinsonian symptoms were reported. Splenic enlargement, with or without hepatomegaly was presented in four cases, whereas bone disease and thrombocytopenia occurred in two. Moreover one case presented with pancytopenia, paraproteinemia and possible myoclonic epilepsy. The severity score index [4] ranged from 4 to 10.

### 3.3 In silico analysis of effects of novel GBA1 mutations

While the splice site mutation would clearly indicate loss of function allele, the four missense mutations raise further questions as to their structural effects on glucocerebrosidase function. The effects of the missense mutations have been mapped onto the 3D structure of the GBA1 protein in Fig. 1 of Supplementary data by the viewer *Protein Workshop* [5]. We have since analyzed their effects on protein stability and malfunction by the *Site Directed Mutator* (*SDM*) bioinformatics tool [6] and the *HOPE* web server [7].

Although A341V was predicted to be stabilizing, the mutant residue in the protein core was larger and could disrupt the local structure surrounding the active site. This residue together with the Asp127, Glu235, Glu340 and Asp380 residues has been located in the GBA1-substrate interaction region and participates in the correct catalytic processing of the substrate [8]. Another mutation located in the same amino acid position, A341T, was previously reported in a Type III GD patient of Finnish origin, with L444P as second allele [9] and in a Type I GD patient of Swedish ancestry, combined with N370S [10]. Both R262G and V447E were predicted to be highly destabilizing and cause protein malfunction. The loss of positive charge in R262G is likely to affect the interactions of the protein with other molecules. Another amino acid change in the same position, R262H, has been linked to PD by the Leiden Open Variant Database [11] and has been found in multiple system atrophy [3] but was not previously found in GD. Related to V447E the mutant amino acid was predicted to alter the β-sheet secondary structure found in that region. Also its negative charge is likely to cause loss of hydrophobic interactions with other molecules. In this sense, the residue has been located in the Saposin C interaction surface and contacts Ala446, involved in the proper structural arrangement of the surrounding area [8]. It is likely that the loss of hydrophobic environment damages the binding site for the Saposin C. These results were also supported by the predictions of the tools *SIFT* [12] and *PolyPhen* [13], which indicated that all four newly identified missense mutations are likely to be pathogenic (Table 2, Supplementary data).

## 4 Discussion

Here we report a comprehensive genotypic analysis of the *GBA1* mutation spectrum in 73 Type I GD patients using Sanger sequencing technology. As predicted from numerous studies, N370S was the most common mutation; it accounted for nearly 50% of the mutant *GBA1* alleles, and much greater than that reported (29%) in a smaller study reported by Hatton et al. [14] in 46 British and Irish patients with GD. This might be explained by the fact that just 22% of patients in our study were Ashkenazim. Ethnic origin has a large effect on the distribution of mutations, being particularly evident for N370S, which is not generally prevalent in the Chinese population [15]. The Turkish population has an N370S allele frequency over 30% [16] and the frequency in Iberian populations are 50.2 (Spanish) and 53.7%, (Portuguese), respectively [17,18] almost identical to our cohort.

The number of mutations in *GBA1* reported in GD is now about 220 with about 20 variants reported as non-pathogenic variants [19,20]. Both variant types occur throughout the molecule. It is not clear whether each of the reported pathogenic variants is complete loss of function alleles and this is likely to influence the degree to which they increase the risk of PD or if other distinct effects on lysosomal machinery are also contributing. The alleles we report here clearly reduce enzymatic activity sufficiently to induce enzyme deficiency since they cause GD in combination with a second *GBA1* mutant allele. Although further functional analysis of these mutations will be needed to elucidate completely the biochemical processes underlying the disease, they might substantially increase the risk of developing parkinsonism. In a previous phenotyping study, we identified an individual carrying the G250V mutation in heterozygosity and who had clinical features of early PD [21]. We report here four new missense mutations and one splice site mutation which are clearly loss of function alleles and which may, therefore, be suspected of harbouring a predisposition to PD.

## Author roles

Dr Raquel Duran carried out the laboratory work and wrote the first draft of the paper, Dr Alisdair McNeill assessed the patients and edited the manuscript, Prof Atul Mehta, Dr Derralyn Hughes, Prof Timothy M. Cox, identified the Gaucher's patients and criticised the manuscript. Prof Anthony H.V. Schapira conceived of the overall study, obtained the funding to support it and criticised the manuscript, Prof John Hardy supervised the molecular work, obtained the funding and co-wrote the first draft of the manuscript.

## Financial disclosures and support

Dr Raquel Duran has received a fellowship from the Alfonso Martin Escudero Foundation (Spain). Dr Alisdair McNeill is funded by a UK Medical Research Council research training fellowship. Prof Atul Mehta has received research grants, honoraria for speaking and advisory boards from Genzyme, Shire and Actelion. Dr Derralyn Hughes has received research grants, honoraria for speaking and advisory boards from Genzyme, Shire and Actelion. Prof Anthony H.V. Schapira receives research support from the UK Medical Research Council, the Wellcome Trust, the Parkinson's disease UK and the Kattan Trust. Prof John Hardy receives research support from the UK Medical Research Council, the Wellcome Trust, the Parkinson's disease UK and the Brain Research Trust.

The work received substantial support by Wellcome Trust/MRC Joint Call in Neurodegeneration Award (WT089698) to UCL, the MRC Unit in Dundee and the University of Sheffield.

The following are the supplementary data related to this article.

**Supplementary Fig. 1:**
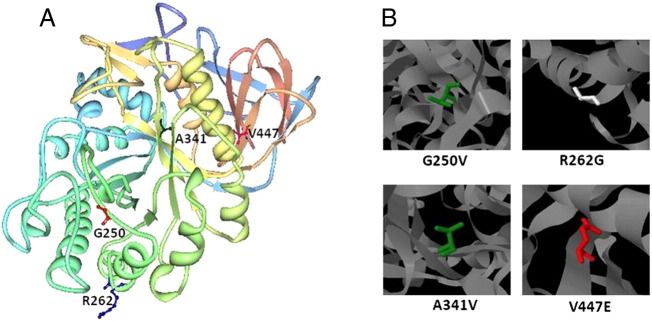
Distribution in the three-dimensional structure of GBA1 of the novel mutations (A) Position of the wild-type residues whose amino acid changes lead to novel mutations. (B) Structural modifications of the mutant residues on GBA1.

Supplementary Table 2 PolyPhen and SIFT scores and prediction results of the novel *GBA1* variants on the protein function.

## Figures and Tables

**Table 1:** Clinical features of the subjects carried the novel mutations.

Subject	Genotype	Clinical features			
		Age	Sex	Symptoms^a^	SSI
GD1	N370S/IVS9+1	30	Male	HSP; T (26)	6
GD2	R262G/RecNcil	51	Male	SP; P; PA; ME (47)	8
GD3	A341V/N370S	26	Female	SP; T (15)	4
GD4	N370S/V447E	55	Female	S; H; BD (6)	10
GD5	N370S/IVS9+1	70	Female	BD	NA
GD6	N370S/G250V	45	Female	NA	NA

GD: Gaucher's disease patient; SSI: severity score index; NA: not available; HSP: hepatosplenomegaly; T: thrombocytopenia; SP: splenomegaly; P: pancytopenia; PA: paraproteinemia; ME: myoclonic epilepsy; S: splenoctomy; H: hepatomegaly; BD: bone disease.

a Numbers in brackets mean age in what symptoms were presented.
