# Supplementary material for: Novel pathogenic mutations in the glucocerebrosidase locus
Source: Mol Genet Metab. 2012 Aug;106(4-2):495–7. doi: 10.1016/j.ymgme.2012.05.006 (PMC3426931; doi:10.1016/j.ymgme.2012.05.006)
Supplement: Supplementary Table 2 — PolyPhen and SIFT scores and prediction results of the novel GBA1 variants on the protein function. [file mmc1.doc]

## Supplementary data

## Table 2 PolyPhen and SIFT scores and prediction results of the novel *GBA1* variants on the protein function

| **Amino acid position** | **Amino acid reference** | **Amino acid substitution** | **PolyPhen** | | **SIFT** | |
| --- | --- | --- | --- | --- | --- | --- |
|  |  |  | **Score^a^** | **Prediction** | **Score^b^** | **Prediction** |
| 250 | G | V | 1.647 | Probably  damaging | 0.34 | Tolerated |
| 262 | R | G | 1.694 | Possibly damaging | 0.37 | Tolerated |
| 341 | A | V | 1.963 | Possibly  damaging | 0 | Damaging |
| 447 | V | E | 1.912 | Probably  damaging | 0 | Damaging |

^a^ PolyPhen computes the possible impact of an amino acid substitution (AAS) on the structure and function of human proteins. Predictions are assigned as (1) probably damaging, with a score ≥ 2.00 (supposed to affect protein function or structure with high confidence); (2) possibly or probably damaging, depending on substitution site properties, with a score between 1.50-1.99 (supposed to affect protein function or structure) and (3) benign, with a score ≤ 0.50 (most likely having no phenotypic effect).

^b^ SIFT scores range from 0 to 1 and represent the scaled probability of an AAS to be tolerated. AASs with scores that either math with or fall below 0.05, are predicted to affect protein function and are considered as damaging or intolerant. While that AASs with scores ≥ 0.05 are predicted to be tolerated.
